# Virtual Reality (VR)-Based Environmental Enrichment in Older Adults with Mild Cognitive Impairment (MCI) and Mild Dementia

**DOI:** 10.3390/brainsci11081103

**Published:** 2021-08-22

**Authors:** Waleed Riaz, Zain Yar Khan, Ali Jawaid, Suleman Shahid

**Affiliations:** 1 Computer Human Interaction and Social Experience Lab (CHISEL), Syed Babar Ali School of Science and Engineering (SBASSE), LUMS University, Lahore 54972, Pakistan; 17030062@lums.edu.pk; 2 Medical College, Aga Khan University, Karachi 74800, Pakistan; yarkhanzain@gmail.com; 3 Laboratory for Translational Research in Neuropsychiatric Disorders (TREND), BRAINCITY: Centre of Excellence for Neural Plasticity and Brain Disorders, Nencki Institute of Experimental Biology, 02-093 Warsaw, Poland

**Keywords:** virtual reality (VR), environmental enrichment, dementia, mild cognitive impairment (MCI), tolerability, interactivity, cognition, mental wellbeing

## Abstract

Background: Despite an alarming rise in the global prevalence of dementia, the available modalities for improving cognition and mental wellbeing of dementia patients remain limited. Environmental enrichment is an experimental paradigm that has shown promising anti-depressive and memory-enhancing effects in pre-clinical studies. However, its clinical utility has remained limited due to the lack of effective implementation strategies. Objective: The primary objective of this study was to evaluate the usability (tolerability and interactivity) of a long-term virtual reality (VR)- based environmental enrichment training program in older adults with mild cognitive impairment (MCI) and mild dementia. A secondary objective was to assess the effect of VR-based environmental enrichment on stabilization of cognitive functioning and improvement of mental wellbeing in older adults with MCI and mild dementia. Methods: A total of seven participants (four patients with MCI and three with mild dementia) received biweekly VR-based environmental enrichment over a course of 6 months. The tolerability and interactivity of the participants in the VR training was serially assessed via virtual reality sickness questionnaire (VRSQ) and recording of input-error ratio. Cognitive functioning was assessed through Montreal cognitive assessment (MoCA) before and after the study. Mental wellbeing was assessed through Warwick-Edinburgh Mental Well Being Scale (WEMWBS). Results: VR-based environmental enrichment was well-tolerated by the patients with significant decrease in VRSQ scores (*p* < 0.01) and input-error ratio (*p* < 0.001) overtime. VR training was also effective in stabilization of MoCA scores over the course of therapy (non-significant difference in the MoCA scores before and after the therapy) and was associated with a trend (*p* < 0.1) towards improvement in WEMWBS scores between the first and the last assessments. Qualitative observations by the care-givers further corroborated a noticeable improvement in mental wellbeing of patients. Conclusions: This pilot study shows that VR can be a feasible, tolerable, and potentially effective tool in long-term support of older adults with MCI and mild dementia.

## 1. Introduction

The global prevalence of dementia is projected to increase to 82 million by 2030 according to the estimates of World Health Organization. Dementia is often preceded by a condition known as mild cognitive impairment (MCI). MCI is characterized by subtle cognitive deficits that limit the activities of daily living and is considered an intermediary stage between normal cognition and dementia [1]. 

Due to the alarming rise of dementia and MCI globally and owing to the lack of efficacious pharmacological therapies for these conditions, there has been a rising interest in development of non-pharmacological interventions that can support these patients. Several such interventions target stimulation of specific cognitive domains. For instance, reminiscence therapy prompts recall of memories associated with events, people, and places [2]. Similarly, reality orientation therapy involves reinforcement of orientation information to help dementia patients feel a greater sense of control [3]. Furthermore, several mobile applications are now in use to help dementia patients with reminders [4], creative thinking [5], functional independence [6], and activity recognition [7].

Environmental enrichment (EE) is a pre-clinical modality that carries remarkable potential for preventive and therapeutic use in dementia [8,9]. EE has been widely used in laboratory animals and combines cognitive stimulation, physical activity, and increased interaction of the animals with their surroundings. EE enhances plasticity and its effects in preventing cognitive loss induced by aging and neurodegeneration are well-documented in animal models [9,10,11]. However, the clinical use of EE in neurological and psychiatric patient-care settings has remained limited [12]. The recent advent of virtual reality (VR) technology provides a novel approach to implement EE in clinical settings whereby cognitive functioning of dementia patients could be improved through combining elements of cognitive stimulation with virtual immersion. 

VR has been used previously in dementia patients (Table 1). Moyle et al. investigated the effectiveness of a virtual reality forest in increasing engagement and improving apathy in dementia patients [13]. The participants were found to be more immersed and alert in the virtual environment (VE); however, they also reported increased fear and anxiety. Tabbaa et al. explored the effects of VR based intervention in institutionalized dementia patients. The participants who had limited access to the outside world due to their physical and mental disability were provided a virtual view of five different natural environments. Besides high levels of immersion and engagement, positive effects on patient wellbeing were also recorded [14]. Park et al. conducted a randomized control trial to compare the effectiveness of virtual reality-based cognitive-motor rehabilitation (VRCMR) with conventional cognitive rehabilitation (CCR) in patients with MCI. The trial demonstrated that VRCMR can enhance motivation for rehabilitation and cognitive function in older adults with MCI better than CCR [15]. However, it is not clear if the improvement in cognitive functioning in this study is due to use of VR-based immersive environment or incorporation of motor rehabilitation in the VRMCR programs. 

While these studies provide some preliminary evidence for the potential of VR-based interventions in dementia patients, there is a clear need for further investigations. The previous studies have been limited in scope, assessed isolated cognitive domains usually for durations of less than two weeks and have not focused on EE. This necessitates future studies on the long-term tolerability and effectiveness of VR in dementia and MCI that are based on longitudinal assessments and incorporate the stimulatory nature of EE. We performed a pilot study of VR-based EE in older adults with MCI and mild dementia with an aim to investigate the usability of VR, as well as its longitudinal effect on cognitive performance and mental wellbeing of the participants.

## 2. Methods

### 2.1. Study Design

#### 2.1.1. Equipment & Software

VR equipment comprised HTC Vive [16] with two displays of 1080 × 1200 pixels each with a viewing angle of 110 degrees tethered to a high-powered PC, two base stations for movement tracking, and two HTC Vive motion controllers for input. A screen was used to duplicate the subject’s view that could be observed by the caregivers and the research team. The participants could freely move around in a 30 m × 40 m room that was specifically designed to allow for unobstructed movement within the VEs. The assets for the VEs were created in Autodesk Maya and 3DS Max and assembled in Unity 3D using Visual Studio. Reference images were used to ensure realism and accuracy in creating VEs. 

#### 2.1.2. Virtual Environments (VEs) Design

VEs were specifically designed to stimulate several cognitive domains that are impaired in dementia via enhanced engagement ensured through virtual immersion in a familiar environment and gamification of the cognitive tasks. Furthermore, VEs were designed to motivate participants to complete the tasks while stimulating working memory, short-term memory, attention, orientation, recognition, and executive functioning, different cognitive domains that can be clinically assessed [17,18,19]. 

The VEs were selected to depict real-world landmarks with high likelihood of familiarity for the participants. The three VEs that were created for this study comprised virtual versions of (1) the Great Wall of China, (2) the Grand Mosque in Mecca and (3) the Pyramids of Egypt. Familiar surroundings were used to instill a sense of comfort among the participants that could also help in reminiscence by re-enacting existing associations [20,21]. Special attention was paid to include notable real-life details in VEs to increase engagement. For example, VEs had inter-changeable graphics including animals, day/night cycles and terrain artifacts like mountains. This ensured that the patients had new things to observe at each session. 

The tasks were specifically designed to stimulate different cognitive domains, such as registration, short-term memory, attention, alertness, orientation to time and space, working memory, visuospatial navigation, motor coordination, and executive functioning, such as abstraction, calculation and decision making. Furthermore, the tasks were designed to stimulate dopaminergic signaling pathways relevant to reward-seeking and motivation. All these tasks were designed through consultation with a medical doctor/neuroscientist who also clinically examined the patients (AJ). 

**Figure 1:**
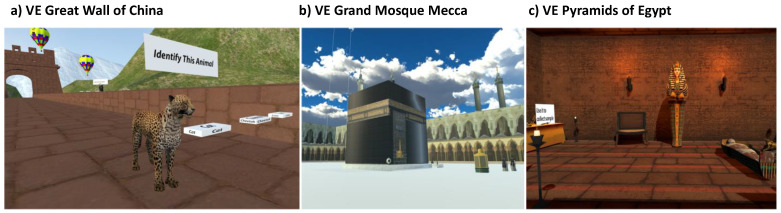
Representation of different virtual environments with examples of environmental enrichment exercises incorporated.

The VE with the Great Wall of China included the following tasks: (a) approaching and recognizing animals for stimulation of visuospatial navigation, goal-directed behavior and recognition; (b) quiz for animals encountered during task a to stimulate short-term memory; (c) targeting static objects in the sky to stimulate motor coordination and goal-directed behavior; and (d) upon successful completion of task c, targeting moving objects in the sky to further stimulate motor coordination and goal-directed behavior (Figure 1a).

The VE with the Grand Mosque of Mecca included the following tasks: (a) approaching and recognizing important land marks in or around the mosque for stimulation of visuospatial navigation, goal-directed behavior, and recognition memory; (b) quiz for landmarks encountered during task a to stimulate short-term memory; (c) counting of minarets around the mosque to stimulate executive functioning; and (d) stating the time shown by the clock tower followed by quiz on relevance of time for prayer timings to stimulate attention, abstraction, and working memory (Figure 1b). 

The VE with the Pyramids of Egypt included the following tasks: (a) recognition of the time of the day by observing the sky and shadows to reinforce orientation; (b) counting of statutes and fire-pits followed by a quiz to stimulate short-term memory and executive functioning; (c) opening of portals and/or revealing artifacts through pressing levers or opening cabinets to stimulate abstraction and executive functioning; and (d) matching shapes to stimulate visual memory and executive functioning (Figure 1c). 

### 2.2. Participants

A total of 8 participants, all above the age of 60 years with subjective memory complaints for at least 3 months, were recruited for the study and assessed at baseline through Montreal cognitive assessment (MoCA) by one of the study investigators and a medical doctor (AJ). All participants were residents of Lahore (Pakistan), were living with their family, and had a minimum of 14 years of education. MoCA scores of the participants were then confirmed by an independent medical doctor blinded to the study design. The participants (*n* = 4) were categorized as MCI if their MoCA score was in the range 24–26 or mild dementia if their MoCA score was in the range 21–23 [19]. The exclusion criteria of the study were, (1) age below 60 years, and (2) reversible causes of cognitive impairment (such as Vitamin B12 deficiency or hypothyroidism), (3) occurrence of a condition that could significantly alter cognitive functioning of the participants, such as stroke, traumatic brain injury, or development of new neurological condition during the study. One participant opted out of the study after initial agreement, citing concerns regarding the utility of VR. The demographic details of the 7 participants who completed the study are present in Table 1. 

### 2.3. Study Procedures

All the participants received VR-based EE on individual basis through sessions conducted every 2 weeks over a period of 6 months. In each session, the participant could select two out of the three VEs for exploration and cognitive training. Before each session, the participants were appraised about the study procedure and their consent to participate in the study was confirmed. The participants were then equipped with the head-mounted display and the hand-held controllers and were immersed virtually in the enriched environment. Once immersed, the participants received audio and visual instructions to perform different cognitive tasks, and their task performance was recorded. Each session lasted 10 min and each VE incorporated 4 cognitive tasks. After the first VR training, the participants were seated and evaluated for any discomfort induced by the VR. The patients could then select the second VE for VR-based EE, and same procedures as in the first training were followed. 

### 2.4. Assessment Tools

The usability of the VR system for EE training in older adults with MCI and mild dementia was studied through testing participants’ tolerability and interactivity during the VR sessions. Tolerability was evaluated through serial VRSQ assessments, whereas interactivity of the participants in VR was ascertained based on the input-error ratio of the participants while navigating through the VEs. 

The Virtual Reality Sickness Questionnaire (VRSQ) assesses 9 different symptoms indicating discomfort that a person can experience while using VR environment, i.e., general discomfort, fatigue, eyestrain, difficulty focusing, headache, fullness of head, blurred vision, dizziness and vertigo [22]. The presence and severity of these symptoms are rated by the participants on a Likert scale (0 = none; 1 = slight; 2 = moderate and 3 = severe). VRSQ evaluation was performed for each participant after the first VR training on each session. 

Input-error ratio was calculated based on the accurate use of the hand-held controllers by the participants. Each hand-held controller had two buttons; one for teleportation to the cursor visible in the VE and the other for motoric actions (for example; pressing the lever, shooting balloon, picking the answer during the quiz). The input-error ratio was calculated based on the number of times a participant erroneously pressed a button divided by the overall use of the two buttons. For the quiz questions, a participant had two trials to pick the right answer, and only the second wrong answer was counted as an error. The participant was then redirected to the next quiz question or task. 

Montreal cognitive assessment (MoCA) test was used to assess cognitive functioning. MoCA is a 30-point test administered in approximately 10 min and assesses several cognitive domains; (1) visuospatial and executive functioning, (2) naming, (3) memory (working), (4) attention, (5) language, (6) abstraction, (7) delayed recall, and (8) orientation [19]. MoCA assessment was performed at baseline and upon completion of the study by an unblinded investigator. However, the scores were confirmed by a medical doctor blinded to the study design. 

The mental wellbeing of the participants was assessed through the Warwick-Edinburgh Mental Well Being Scale (WEMWBS) [23]. The WEMWBS is a 14-item scale that assesses patients’ subjective feeling of wellbeing scored over a 5-point Likert-like scale. The WEMWBS was performed at the end of each session starting from the 7th session after it was ensured that the patients had stopped experiencing any discomfort induced by VR to preclude any bias induced by the participants’ current mood at the time of evaluation. 

### 2.5. Statistical Analysis

All statistical analyses were performed using GraphPad Prism Version 9. Numerical VRSQ scores and the input-error ratio were compared across different sessions using repeated measures one-way ANOVA, whereas Dunnett test was used for post hoc analysis to compare scores during the first session with all subsequent sessions. MoCA scores and WEMWBS between the first and the last assessments were compared through paired *t*-test. The error bars in the figures indicate standard error means. 

### 2.6. Ethical Considerations

Informed written consent was taken from both the participants, as well as their accompanying family member before the start of the study and verbally confirmed at each subsequent session. The consent forms were prepared according to the National Health Care Bill 2017 of the Government of Pakistan [24]. A “Code of Ethics” contract was also signed as per Pakistan Medical and Dental Council (PMDC) requirements to ensure the confidentiality of all medical records [25]. The study procedures were approved by the Intuitional Review Board, Syed Babar Ali School of Science and Engineering, LUMS University for the IRB titled ‘Using virtual reality (VR) based interactive systems to improve the well-being of people with dementia’ dated June 2018. 

## 3. Results

**Figure 2:**
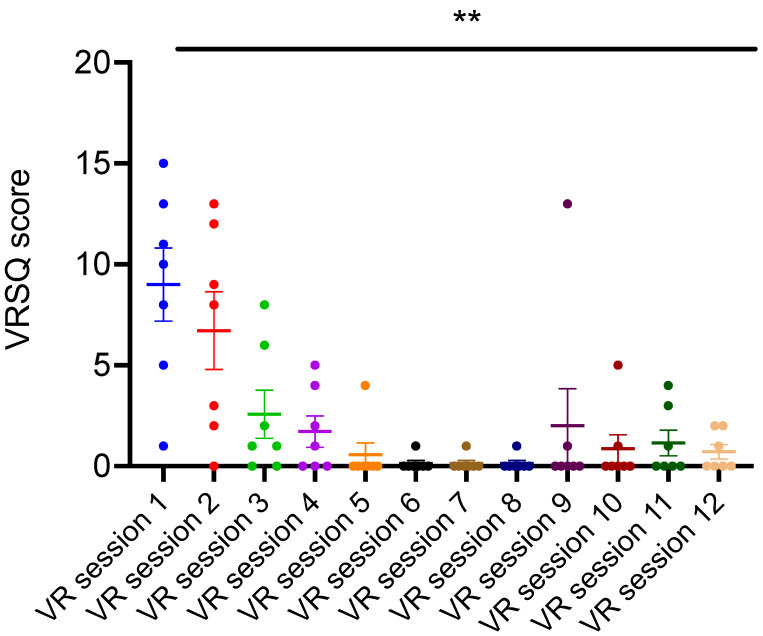
Participants’ tolerability to VR-based environmental enrichment as assessed by serial VRSQ scores. VRSQ scores of the participants across the 12 VR sessions significantly decreased in comparison to the first session (One-way repeated measures ANOVA; F = 9.18; ** indicate *p* < 0.01; *n* = 7; post hoc comparisons: VR session 1 vs. VR sessions 3,4,5,6,7,8,10,11,12 *p* < 0.05; VR session 1 vs. VR sessions 2, 9 *p* > 0.05). Error bars indicate standard error means.

**Figure 3:**
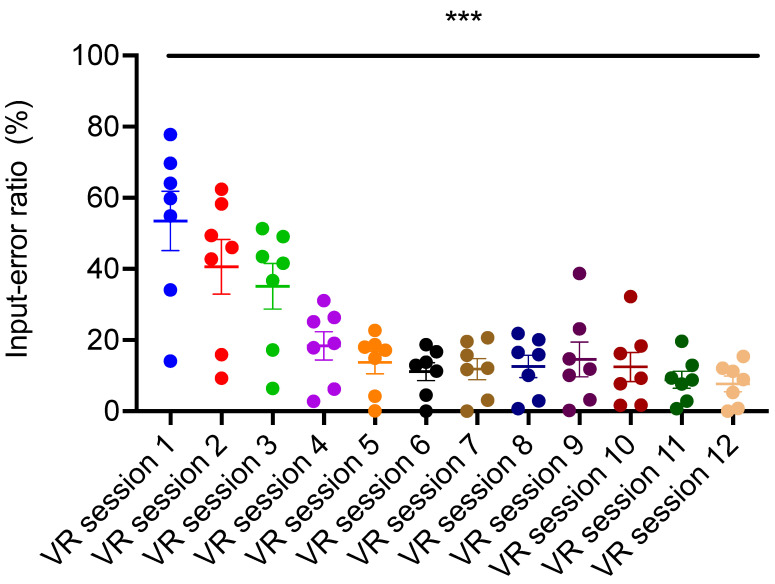
Participants interactivity in the VR assessed via recording of Input-error ratio. Input-error ratio of the participants across the 12 VR sessions significantly decreased in comparison to the first session (One-way repeated measures ANOVA, F = 24.98; *** indicate *p* < 0.001; *n* = 7, post hoc comparisons: VR session 1 vs. VR sessions 2,3,4,5,6,7,8,9,10,11,12 *p* < 0.05). Error bars indicate standard error means.

The overall scores for VRSQ parameter significantly (*p* < 0.01) decreased over time (Figure 2) indicating that VR-based EE was well-tolerated by the participants. Similarly, input-error ratio significantly (*p* < 0.001) decreased over time (Figure 3) as dynamic adjustments were made to the VR controller devices, rendering them easier to use by the participants. These results suggest that navigation and task-completion in VR-based EE training was easy to learn by the participants and well-tolerated. 

**Figure 4:**
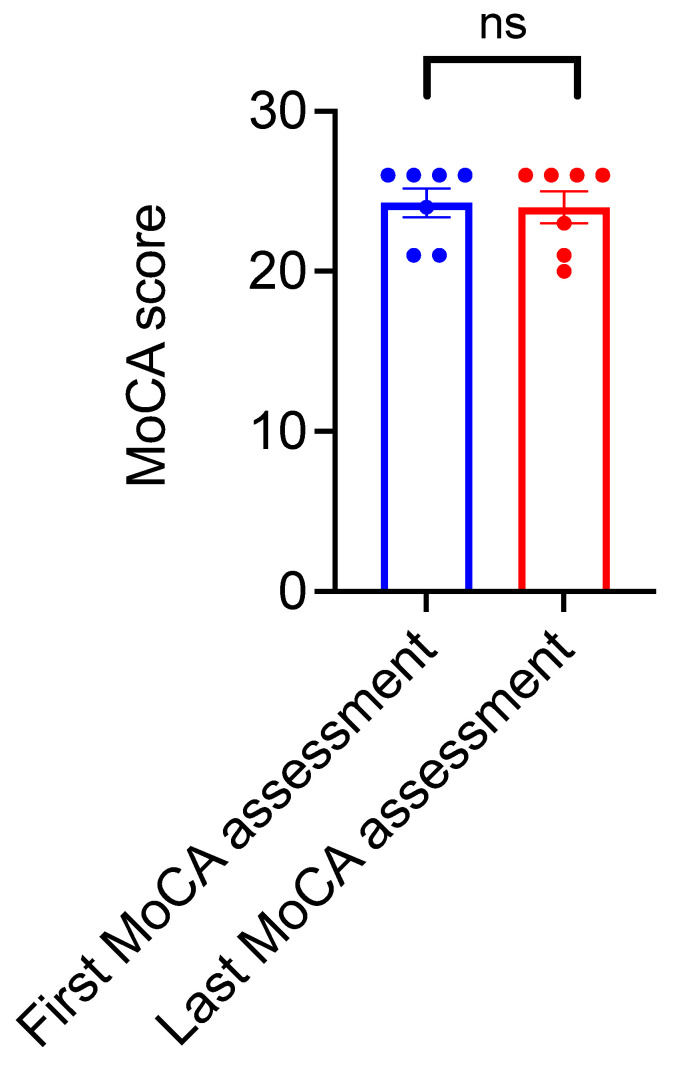
Assessment of participants’ cognitive functions over the course of VR-based environmental enrichment training based on Montreal cognitive assessment (MoCA). MoCA scores of the participants before (first MoCA assessment) and after (last MoCA assessment) the VR-based environmental enrichment training over 6 months do not significantly differ (Paired *t* test, *t* = 1.55, df = 6, ns indicates *p* = 0.1723, *n* = 7). Error bars indicate standard error means.

Cognitive functioning of the participants was assessed through MoCA prior to the first VR session and after the last VR session. A comparison of the two assessments showed stabilization of MoCA scores, as the mean MoCA scores between the two assessments performed prior to and after the VR-based training did not differ significantly (Figure 4).

**Figure 5:**
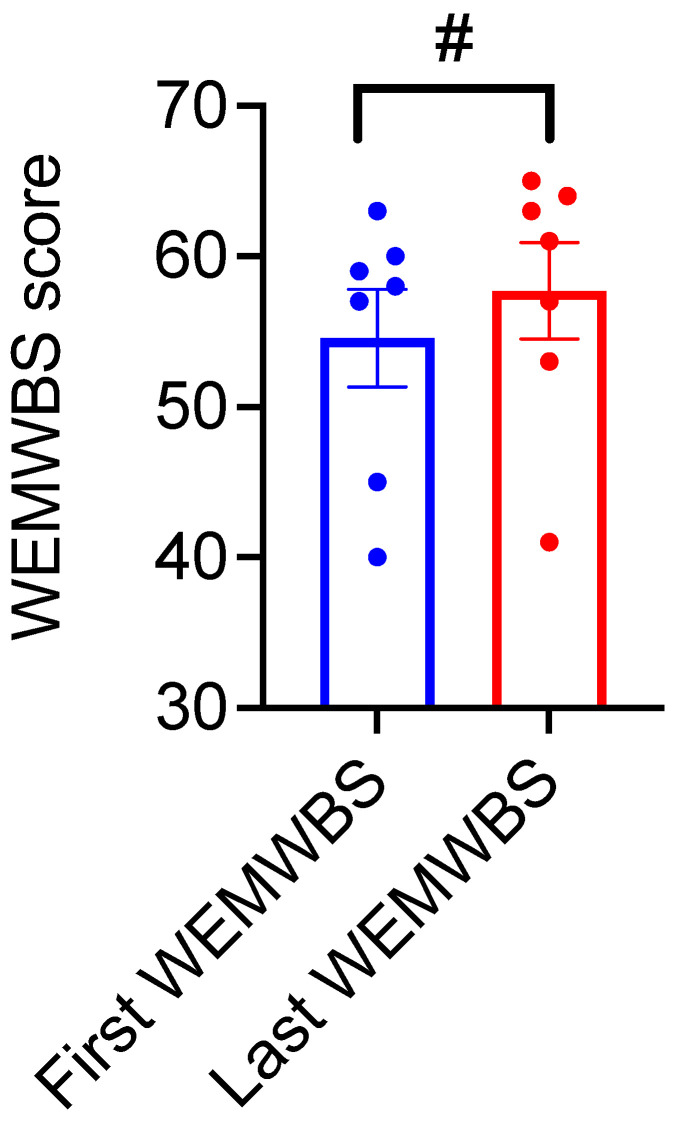
Mental wellbeing of the participants assessed via Warwick-Edinburgh Mental Wellbeing Scale (WEMWBS). WEMWBS scores of the participants at the last WEMWBS assessment show a trend towards increase compared to the first WEMWBS assessment (Paired *t* test, *t* = 2.27, df = 6, # indicate *p* = 0.0641, *n* = 7). Error bars indicate standard error means.

WEBWMS assessments were performed 3 months after the start of VR sessions after ensuring that the patients had achieved good tolerability of the VR. Participants showed a trend (*p* < 0.1) towards improved mental wellbeing between the first and the last WEBWMS assessments (Figure 5). In addition to WEBWMS, several qualitative observations from the care-givers were received during the VR sessions, indicating an overall improvement in patients’ reminiscence, mood, engagement, and procedural skills (Appendix A Section: Qualitative Observations). 

## 4. Discussion

The results of this longitudinal pilot study indicate that VR is potentially feasible and well tolerated as a long-term supportive modality in MCI and mild dementia. Importantly, VR can provide an engaging and immersive approach for implementing EE in humans. Besides the gradual significant reduction in the input-error and discomfort related to VR, the incorporation of EE paradigms in VR led to a non-significant increase in participants’ mental wellbeing and a stabilization of their cognitive functioning. Additionally, VR-based EE was subjectively viewed by the caregivers/family members as a means to increase patients’ attention, focus, presence, engagement, and motivation. 

VR is now increasingly recognized as a technology that has the potential to support dementia patients. A comprehensive review of existing literature reveals at least 20 studies that have tested VR on persons with dementia and/or MCI (Table 2). The scopes of these studies have been variable ranging from VR-based training for enhancing memory retention, promoting reminiscence, improving balance, and mental wellbeing. However, several studies comprised only single sessions or assessed isolated cognitive domains. Notably, very few studies evaluated the tolerability of VR in dementia patients and none employed VRSQ for assessment of tolerability. VRSQ is a validated and reliable tool to assess discomfort induced by immersion in VR environments and is considered superior over the previously used simulator-sickness questionnaire that does not include the unique attributes of VR immersion [26]. Similarly, the feasibility of VR use was not systematically evaluated in most of the studies. Serial longitudinal assessments of tolerability and interactivity, interval assessments for cognitive functioning and mental wellbeing, and use of culturally relevant and familiar immersive environments are important attributes of our study. Considering the special needs of the dementia patients, the VR set-up was dynamically optimized to increase usability. The VEs as well as the tasks incorporated within them were specifically designed to have contextual relevance, so patients were able to relate with these activities and felt comfortable in exploration. In future studies, it will be interesting to incorporate tasks that are more relevant to activities of daily living (ADLs); such as grooming, cooking, ironing clothes, brushing teeth, etc., to evaluate the efficacy of incorporating ADLs into VR-based EE for older adults with MCI and dementia. 

The most prominent aspect of this study is incorporation of EE in the VR setting. Previous attempts to translate EE from pre-clinical to clinical settings have been limited by several factors. Among those, lack of standardization of stimulatory activities and variability in patient motivation have been cited as strong hurdles in implementing EE in the clinical settings [12]. Gamification of EE activities through immersion in VR in this study provided a standardized way of enrichment and engagement for the patients without a compromise on patient autonomy. Furthermore, the use of reminiscing stimuli was incorporated to induce a state of plasticity in the brains, potentially rendering them more conducive to the effects of enrichment. Our study does not reveal a concrete mechanistic explanation of how VR-based EE could stabilize cognitive functioning in older adults with MCI and/or mild dementia. Considering that the sessions were performed biweekly, it is plausible that the effects were mediated by improvement in participants’ behavior or motivation during the intervening periods. Indeed, qualitative observations from the care-givers (Appendix A section) support this possibility, which warrants standardized examination in future studies. 

However, this study also has several limitations that warrant careful consideration while interpreting the results. First, considering the novelty of the modality and the trial nature of the study, the recruitment of patients was considerably challenging. This resulted in a very small sample size. A post hoc power analysis based on a 6% prevalence of dementia in population above 65 years in Pakistan reveals that the margin of error could be as high as 16% in interpreting the results of our study [45]. Additionally, the gender distribution of the study participants was skewed as there were more male patients than female ones. Another critical point to consider is the lack of appropriate control groups in the study. The unique characteristic of this study required at least two control groups; (1) a matched cohort that would not receive any VR-exposure, and (2) a matched cohort that would be exposed to VR but would not receive any VR-based EE or stimulation. Considering the trial nature of the study, recruitment of these control groups could not be achieved. Furthermore, it is important to consider that the results of the study may also be influenced by various confounding factors, i.e., the presence of co-morbidities, the nature of care facilities at participants’ residences, level of education and varying levels of disease severity. However, we tried to reduce these influences by ensuring that all participants had either MCI or mild dementia, were residing with their families, and had minimum 14 years of education. 

Future efforts on using VR-based EE strategies could benefit substantially from larger sample sizes, use of appropriate control groups, and stringent matching and randomization of the participants. It would also be relevant to standardize VEs for broader use and assess the effects of VR-based EE on other measures of quality of life (QoL). Furthermore, the effects of VR-based EE on disease progression should be investigated through brain imaging, such as MRI and/or PET amyloid scans, etc. Finally, mechanistic insight into the potential efficacy of VR-based EE in improving neurobehavioral functioning of dementia and MCI patients is warranted as it is not clear which aspect of EE lead to improvement in participants’ cognition and behavior. 

## 5. Conclusions

In conclusion, this pilot study shows that VR-based environmental enrichment could be implemented as long-term supportive modality in care of patients with MCI and early dementia that has potential to stabilize cognitive functioning of patients as well as improving their mental wellbeing. 

## Figures and Tables

**Table 1:** Demographic details of the participants and their baseline cognitive (MOCA) scores.

Persons with Mild Dementia	**Age**	**Gender**	**MOCA Score (30)**
	72 y	M	21
	65 y	F	24
	69 y	F	21
Persons with Mild Cognitive Impairment	81 y	F	26
	62 y	F	26
	64 y	F	26
	65 y	M	26

**Table 2:** Existing studies on the use of VR in MCI and dementia patients.

No.	First Author (Year of Publication)	Clinical Diagnosis	Sample Size	Mean Age (Years)	Session Length (minutes)	Time Period of Assessment	Safety or Tolerability Measurement	Use of Culturally Relevant Environments
1	Flynn (2003) [27]	Dementia	10	69.6	20	Single Session	Adapted version of the Simulator Sickness Questionnaire	Yes
2	Man (2011) [28]	Dementia	20	80.3	30	10 sessions over 3–5 weeks	None	Yes
3	Burdea (2015) [29]	Dementia	10	63.4	20–40	Variable sessions over 8 weeks	None	None
4	Lancioni (2015) [30]	Alzheimer’s Dementia	16	83	5	Variable for participants	None	Yes
5	Schwenk (2016) [31]	Mild Cognitive Impairment	12	77.8	45	8 sessions over 4 weeks	Custom User Experience Questionnaire	None
6	Manera (2016) [32]	Dementia and Mild Cognitive Impairment	28	75	20	Single Session	1 Self-report questionnaire	None
7	Moyle (2017) [13]	Dementia	10	89	15	Single Session	None	None
8	Hwang (2017) [33]	Mild Cognitive Impairment	12	74.16	30	20 sessions over 4 weeks	None	None
9	Serino (2017) [34]	Alzheimer’s Dementia	10	86.6	20	10 sessions over 3–4 weeks	None	Yes
10	Mrakic Sposta (2018) [35]	Mild Cognitive Impairment	5	72	40	18 sessions over 6 weeks	None	None
11	Anderson Hanley (2018) [36]	Mild Cognitive Impairment	15	69.4	20–40	Variable sessions over 12 weeks	None	None
12	Wall (2018) [37]	Mild Cognitive Impairment	7	82.8	30–45	Variable sessions over 12 weeks	None	None
13	Liao (2019) [38]	Mild Cognitive Impairment	18	75.5	60	36 sessions over 12 weeks	None	Yes
14	Appel (2020) [39]	Dementia	10	86.5	20	Single Session	None	None
15	Coelho (2020) [40]	Dementia	9	85.6	15	4 Sessions	Simulator Sickness Questionnaire	Yes
16	Park (2020) [41]	Amnestic Mild Cognitive Impairment	10	71.8	30	24 sessions over 12 weeks	None	Yes
17	Jung Yun (2020) [42]	Mild Cognitive Impairment and Mild Dementia	11	72.6	30	Single Session	None	Yes
18	Thapa (2020) [43]	Mild Cognitive Impairment	34	72.6	100	24 sessions over 8 weeks	None	None
19	Ji-Su Park (2020) [15]	Mild Cognitive Impairment	18	75.8	30	30 Sessions over 6 Weeks	None	None
20	Kim (2021) [44]	Dementia	10	85.8	20–30	1–2 Sessions	None	Yes

## Data Availability

Due to confidentiality reasons, the patient data is not deposited on any repository but is available upon request and confidentiality agreement between the requester and the study authors.

## Appendix A

### Appendix A.1. Qualitative Observations

Qualitative data was collected from the observational notes of the care-givers and has been grouped into three main themes: (1) reminiscence using familiarity, (2) mood and wellbeing, and (3) enhancement of precision and procedural skills.

### Appendix A.2. Reminiscence Using Familiarity

‘He fondly talked about the family members and friends that accompanied him on his visit to China many years ago after visiting the Great Wall of China virtually.’

‘She started talking about the history of the pharos and Egyptian culture which she had read in books and seen in movies.’

### Appendix A.3. Mood and Wellbeing

‘She was particularly excited to see the animals. She pointed out and recognized all of them.’

‘She frequently talked about her virtual visit to the Grand Mosque and mentioned how happy and blessed she feels to have experienced it. She added that she would not be able to visit it in real life due to her frail health.’

### Appendix A.4. Enhancement of Precision and Procedural Skills

‘He was very excited about her target practice skills a lot and also mentioned that he now feels motivated to practice shooting balloons in real life too’

‘She usually remained to herself and moved around very little at home. She’s been more active now after exploring and doing tasks in the sessions. She walks around the house more now and is more energized.’
